# A Comparison of Techniques of Internal Jugular Vein Cannulation: Anatomical Landmark, Ultrasound Guided Pre-location, and Real-Time Ultrasound Guided

**DOI:** 10.7759/cureus.54499

**Published:** 2024-02-19

**Authors:** Syed Shabbir Ahmed, Khalid Samad, Muhammad S Yousuf, Muhammad Qamar-ul-Hoda

**Affiliations:** 1 Anaesthesiology, Aga Khan University Hospital, Karachi, PAK; 2 Anaesthesia and Critical Care, Aga Khan University Hospital, Karachi, PAK; 3 Anaesthesia and Critical Care, Aga Khan Health Service, Karachi, PAK

**Keywords:** anatomical landmark, ultrasound, central venous catheter, cardiac anaesthesia, cardiac surgery

## Abstract

Objective: The objective of our study is to compare the success rate, duration, and incidence of complications of a right internal jugular vein (IJV) cannulation by using three different techniques.

Methodology: A randomised controlled trial was conducted at a tertiary care teaching hospital. A total of 201 patients were randomly allocated to one of the following three groups (67 in each group). Techniques were categorised as anatomical landmark technique group (Group ALT), ultrasound guided pre-location group (Group USG-Pre), and real-time ultrasound-guided technique group (Group USG-RT).

Interventions: Central venous catheter insertion via three techniques.

Results: In 138 (73.01%) patients’ IJV canulated in the first attempt, USG-RT, USG-Pre, and ALT were 51 (83.6%), 44 (72.1%), and 43 (64.2%), respectively. On the other hand, 37 (19.57%) patients were required in the second attempt, while only 14 (7.40%) patients were required in the third attempt for successful IJV cannulation. The success rates, as defined in our study, were only 138 (73%) as, in 51 (27%), we cannulated in more than a single attempt or switched to another technique. We found a significant difference in preparation time in all techniques as P-value <0.05, but no significant difference was found in venous access time, cannulation time, and duration of the procedure.

Conclusions: Any technique can be used for IJV cannulation, but the most acceptable is the real-time US technique. However, no difference in the overall procedure time among all three techniques was noted, and no major incidence of complication was found.

## Introduction

In cardiac surgery, as well as in a critical care unit, the insertion of central venous catheters (CVC) is a common practice. It serves a variety of functions, including administering fluids and medications and monitoring central venous pressure (CVP), pulmonary artery catheter insertion, and total parenteral nutrition. It has many complications despite frequent practice and training such as hemothorax, pneumothorax, air embolism, tracheal injury, chylothorax, hydrothorax, arterial puncture, thrombosis, infection, catheter disposition, and even cardiac perforation. These complications usually occur when doctors have little experience and patients have structural abnormalities (such as obesity, tumors, vascular thrombosis, and congenital malformations), emergency conditions, and the existence of comorbidities such as emphysema and coagulopathies, which are the main causes of these problems [1-4].

CVC can be inserted either with an anatomical landmark or ultrasound guided (USG) technique. In 1966, right internal jugular vein (IJV) cannulation was first described by Hermosura et al., and since then, it has grown in popularity as a method of central venous cannulation [5]. In addition, most investigators have shown that the use of ultrasound for IJV cannulation is more successful in terms of less number of attempts, fewer complications, and less time consumption. Both the European and American Society of Anaesthesiology issued guidelines for IJV cannulation and recommended that the ideal technique for adult IJV cannulation is USG. This is true in both elective and emergency clinical settings when CVC installation is required [6,7].

Ray et al. stated that successful IJV cannulation using the anatomical landmark technique was 85% as compared to USG IJV cannulation, which was 95%. The median catheterization time of IJV via USG is shorter than the anatomical landmark technique [8]. The complication rate was 7.5% in the anatomical landmark technique as compared to 2.5% in ultrasound groups. However, another study conducted by Filho et al. showed that the successful cannulation of IJV at the first attempt was 100% via ultrasound guidance as compared to the anatomical landmark technique, which was 60%, but the catheterization time was the same in both anatomical landmark and USG techniques [9]. In terms of complication, there was 0% in ultrasound groups and 2% in anatomical landmark groups. In our region, there was only one study conducted at Military Hospital Rawalpindi by Burki et al. [10]. They compared successful cannulation via ultrasound guidance and anatomical landmark technique and found 100% successful cannulation in the ultrasound group as compared to 92% via the anatomical landmark technique, while the complication rate was 12% in the anatomical landmark group as compared to 0% in the ultrasound groups [10].

Furthermore, with the widespread availability of ultrasound, IJV can be successfully cannulated with a decreasing number of attempts, less time consumption, and a decrease in the incidence of complications. There has been little evidence regarding cannulation of the internal jugular vein under ultrasound guidance at a tertiary care hospital in our population. The primary objective of our study was to compare the success rate of right IJV cannulation by using three different techniques, namely, the anatomical landmark technique group (Group ALT), USG pre-location group (Group USG-Pre), and real-time ultrasound-guided technique group (Group USG-RT). The secondary objectives were to compare the time duration for each technique and the incidence of complications (carotid puncture, hematoma formation, and pneumothorax).

## Materials and methods

After obtaining ERC approval (2019-0781-4270), registration with ClincalTrials.gov (Clinical trial no: NCT05387486), and consent from patients, this randomised controlled trial was conducted at cardiac operating rooms, from August 2019 to July 2021. Inclusion criteria consisted of any patient aged between 18 and 65 years and ASA 3-4 requiring a CVC during surgical procedures coming for cardiac surgery. Anyone who has undergone head and neck surgery in the past and has a head and neck tumor or malignancy, superior vena cava syndrome, coagulopathy, an infection at the cannulation site, previous central venous access, raised intracranial pressure (ICP), emergency surgery, a BMI of more than 30, or patient who refused to cooperate were excluded.

The procedure was performed by either a consultant or a resident who must have passed at least 20 CVC lines via anatomical landmark and 20 via ultrasound guidance. A total of 201 patients, who required CVC monitoring, were included in the study. Patients were randomly allocated to one of the following three groups through the opaque sealed envelope technique (67 in each group). Each envelope contained the name of one technique and was opened before the induction of the patient and the technique assigned to the consultant or resident.

The techniques were categorized as Group ALT, Group USG-Pre, and Group USG-RT. All patients had standard ASA monitoring, including blood pressure, pulse oximetry, and electrocardiogram (ECG), and were induced with standard general anaesthesia for cardiac surgery. All patients were stabilized in the Trendelenburg (20-30°) position after anesthetic induction, with the head tilted slightly to the left. The patient's shoulders were supported by a shoulder roll. An antiseptic solution was used to prepare the right side of the neck area. A trolley for central venous cannulation was prepared by the consultant or resident who performed the procedure.

In Group ALT, anatomical landmarks (sternocleidomastoid muscles, sternal notch, cricoid cartilage, carotid artery, and clavicle) were identified. A 3cc syringe with a 21-gauge needle (locator) was used first to locate IJV immediately lateral to carotid artery pulsation and at the point where the two heads of the sternocleidomastoid muscle form a triangle. After a successful location, an introducer needle of 18 gauge with a 5 mL syringe was put in the same location and connected. The introducer needle was directed towards the ipsilateral nipple at 20-30° with the skin. The ultrasound probe of 4 MHz used in Group USG-Pre was positioned at the level of the cricoid cartilage on the right side of the neck, perpendicular to the skin (out of plane), and connected to the 2D image display of the ultrasound machine M7 system (Mindray, Shenzhen, China, 2014). The IJV and the carotid artery were distinguished by the compressibility of the vein and the presence of pulsations in the artery. The locator needle of 21 gauge was used first to confirm the IJV location. The cannulation or venipuncture was performed at the marked point of the locator needle using an 18-gauge introducer needle. In Group USG-RT, cannulation or venipuncture was performed under USG real-time imaging using an 18-gauge needle with ultrasound guidance. It was proven that the introducer needle had entered the IJV when free-flowing black venous blood returned to the syringe attached to it. The right IJV was then cannulated using Seldingers' approach. The CVC was secured with sutures, and a sterile dressing was applied. The following observations were recorded: number of attempts, venous access time, cannulation time, hematoma formation, arterial puncture, and pneumothorax.

The preparation time was different in three techniques. In the anatomical landmark technique, the preparation time was started from the opening of the sterile CVP pack till the insertion of the locator needle. In USG pre-location and USG real-time techniques, the preparation times were started from the opening of the sterile CVP pack and preparation of the ultrasound probe till the insertion of the locator needle. Venous access times were defined in Group ALT as the time from the insertion of the locator needle till the return of dark-colored venous blood in the attached syringe of the introducer needle. Meanwhile, in both USG techniques, USG-Pre and USG-RT, the preparation time was defined as the time from placement of the ultrasound probe on the neck to locate IJV till the return of dark-colored venous blood in the attached syringe of the introducer needle. In all three techniques, the cannulation time was defined as the time from the return of venous blood in the syringe of the introducer needle till the successful cannulation of IJV. The duration of the procedure was defined in all techniques as the time from the preparation of the CVC trolley till the successful cannulation of IJV. Cannulation was defined as the insertion of the central venous line into the right internal jugular vein. The number of attempts was considered the entry of the introducer needle into the skin and its removal from the skin, which was counted as one attempt. The success of CVC cannulation was defined as the cannulation of the right IJV within the first three attempts, while failure was considered an inability to cannulate the vein by the assigned technique. The position of the tip of the CVC was confirmed by performing a chest radiograph, as per the standard procedure. Complications, if occurred, were managed according to the standard protocol. In case of failure of cannulation by the assigned technique, it was marked as a failure in our study, and the technique through which successful cannulation was done was noted down, and patients were still included in the study.

**Figure 1:**
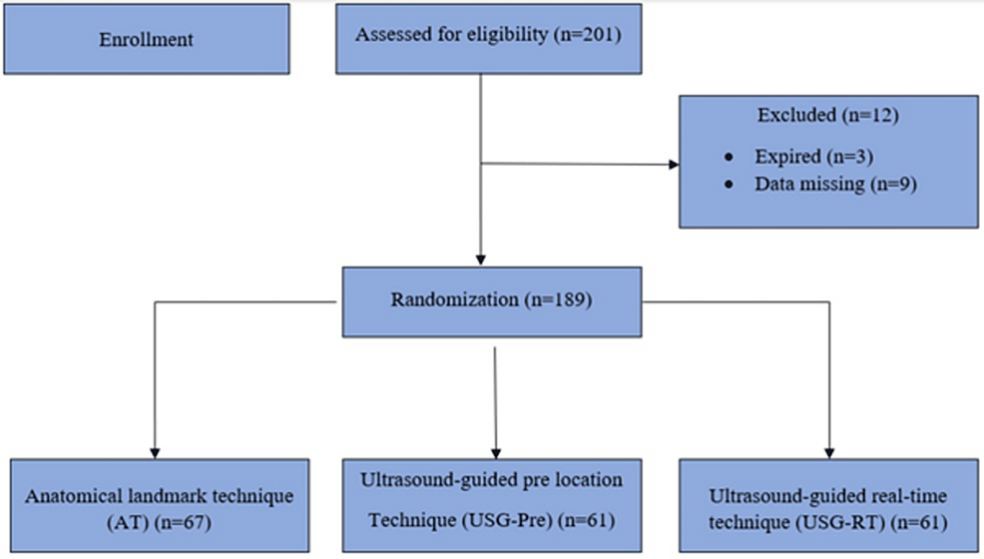
CONSORT diagram of patients having internal jugular vein cannulation

The primary objective of our study was to compare the success rate of right IJV cannulation by using three different techniques, as defined (ALT, USG-Pre, and USG-RT). The secondary objectives were to compare the time duration for each technique and the incidence of complications (carotid puncture, hematoma formation, and pneumothorax). RStudio (version 4.2.2, Posit, PBC, Boston, MA) software was used to calculate the sample size. Based on the previous study conducted in the emergency department at St. Vincent Hospital, Darlington, Australia [11], which revealed a P1=ALT Groups (78.5%), P2=USG Groups (93.9%), and diff=15% (one-sided test), it was estimated that 180 (60 per group) patients were required to achieve 80% power of the test, with a significance level of 0.05. Adjusting for a 10% loss to follow up, around 201 (67 per group) patients were recruited (Figure 1).

All statistical analysis was performed by using Statistical Product and Service Solutions (SPSS, version 19; IBM Corp., Armonk, NY). The success rate was the primary outcome variable, and the techniques of catheterization were an independent variable. Complications, age, gender, weight, and height were also explanatory variables and included in the analysis. Frequency and percentage were computed for categorical variables, while mean and standard deviation were estimated for quantitative variables. The chi-square test or Fisher’s exact test was applied to compare the success rate among groups, while analysis of variance (ANOVA) and the post-hoc test were used to compare mean time differences among groups with significant variables. P<0.05 was considered as significant.

## Results

In this study, male patients were 146 (77%), and female were 43 (22.8%). There were no differences between the groups in terms of age and height, but significant differences were found in weight and BMI among groups (P < 0.05, Tables 1-2). Our year III and IV anesthesia residents were involved in most of the procedures and showed a significant difference, as shown in Table 1.

**Table 1:** Demographic characteristics of the patients according to groups a: ANOVA test, b: chi-square test, c: Fisher’s exact test BMI, Body mass index; ALT, Anatomical landmark technique; USG, Ultrasound guided

Variable	Total (N=189)	Anatomical landmark (N=67)	Pre-location USG (N=61)	Real-time USG (N=61)	P-value
Age (years) ^a^	55.2 (13.4)	57.2 (12.8)	55.0 (13.6)	53.3 (13.6)	0.251
Weight (kg)^ a^	73.0 (14.9)	70.4 (13.6)	71.7 (13.2)	77.2 (17.0)	0.024*
Height (cm)^ a^	164 (7.79)	164 (7.32)	165 (8.60)	164 (7.55)	0.916
BMI (kg/m^2^)^ a^	27.0 (4.91)	26.0 (4.66)	26.5 (4.65)	28.6 (5.14)	0.009*
Gender^ b^					
Male	146 (77.2%)	56 (83.6%)	46 (75.4%)	44 (72.1%)	0.279
Female	43 (22.8%)	11 (16.4%)	15 (24.6%)	17 (27.9%)	
Designation ^c^					
Consultant	9 (4.76%)	3 (4.48%)	1 (1.64%)	5 (8.20%)	0.010*
I	9 (4.76%)	7 (10.4%)	0 (0%)	2 (3.28%)	
II	10 (5.29%)	4 (5.97%)	1 (1.64%)	5 (8.20%)	
III	70 (37.0%)	25 (37.3%)	21 (34.4%)	24 (39.3%)	
IV	67 (35.4%)	18 (26.9%)	31 (50.8%)	18 (29.5%)	
V	18 (9.52%)	5 (7.46%)	7 (11.5%)	6 (9.84%)	
Fellow	6 (3.17%)	5 (7.46%)	0 (0%)	1 (1.64%)	

**Table 2:** Demographic comparison among groups a: ANOVA test, d: Post-hoc test BMI, Body mass index; ALT, Anatomical landmark technique; USG, Ultrasound guided

Variable	Mean ± SD	Pairwise Comparison	Mean ± SD	P-value ^a ^	P-value ^d^
Age (years)	55.2±13.4	Anatomical Landmark vs Pre-location USG	[57.2±12.8] vs [55.0±13.6]	0.251	0.605
		Anatomical Landmark vs Real-Time USG	[57.2±12.8] vs [53.3±13.6]		0.224
		Pre-location USG vs Real-Time USG	[55.0±13.6] vs [53.3±13.6]		0.772
Weight (kg)	73.0±14.9	Anatomical Landmark vs Pre-location USG	[70.4± 13.6] vs [71.7±13.2]	0.024*	0.870
		Anatomical Landmark vs Real-Time USG	[70.4±13.6] vs [77.2±17.0]		0.025*
		Pre-location USG vs Real-Time USG	[71.7±13.2] vs [77.2±17.0]		0.098
Height (cm)	164±7.79	Anatomical Landmark vs Pre-location USG	[164±7.32] vs [165±8.60]	0.916	0.993
		Anatomical Landmark vs Real-Time USG	[164±7.32] vs [164±7.55]		0.952
		Pre-location USG vs Real-Time USG	[165±8.60] vs [164±7.55]		0.914
BMI (kg/m^2^)	27.0±4.91	Anatomical Landmark vs Pre-location USG	[26.0±4.66] vs [26.5±4.65]	0.009*	0.839
		Anatomical Landmark vs Real-Time USG	[26.0±4.66] vs [28.6±5.14]		0.010*
		Pre-location USG vs Real-Time USG	[26.5±4.65] vs [28.6±5.14]		0.053*

**Figure 2:**
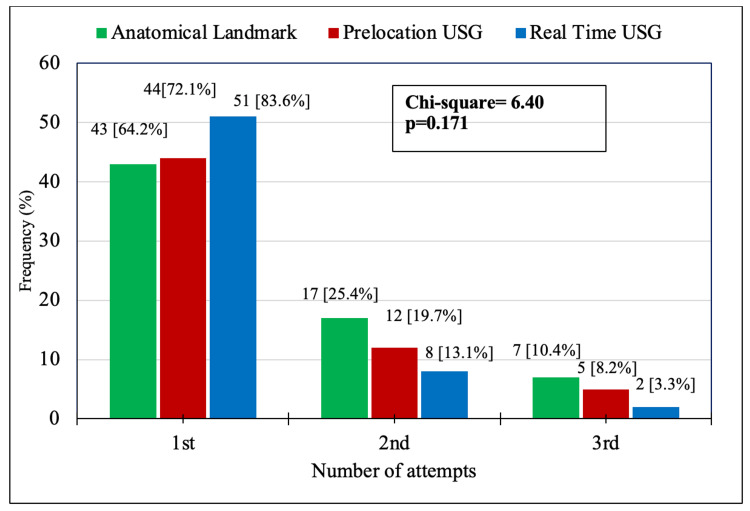
Comparison of the rates of IJV successfully cannulated among groups ALT, Anatomical landmark; USG, Ultrasound guided

In 138 (73.01%) patients IJV canulated in the first attempt, USG-RT, USG-Pre, and ALT were 83.6%, 72.1%, and 64.2%, respectively. On the other hand, 37 (19.57%) patients were required in the second attempt, while only 14 (7.40%) patients were required in the third attempt for successful IJV cannulation, as shown in Figure 2.

We only found difficulty in cannulating a total of 51 (26.98%) patients using the assigned technique; thus, we switched only 14 (7.40%) patients to another technique, but did not switch 37 (19.57%) patients. Hence, IJV cannulation was done in all recruited patients, but the success rate, as defined in our study, was only 73% as, in 27%, we cannulated in more than a single attempt or switched to another technique (Table 3).

**Table 3:** Number of techniques switched in case of failure of internal jugular vein cannulation ALT, Anatomical landmark technique; USG, Ultrasound guided

Number of attempts	Switched or not switched	Anatomical landmark, n=67	Pre-location USG, n=61	Real-time USG, n=61	Total
2^nd^	Total	17 (25%)	12 (20%)	8 (13%)	37
	Not Switched	14 (21%)	9 (15%)	7 (11%)	30 (81%)
	Switched	3 (4%)	3 (5%)	1 (2%)	7 (19%)
	ALT	0	1 (2%)	0	1 (3%)
	USG-PL	0	0	1 (2%)	1 (3%)
	USG-RT	3 (4%)	2 (3%)	0	5 (13%)
3^rd^	Total	7 (10%)	5 (8%)	2 (3%)	14
	Not Switched	5 (7%)	0	2 (3%)	7 (50%)
	Switched	2 (3%)	5 (8%)	0	7 (50%)
	USG-RT	2 (3%)	5 (8%)	0	7 (50%)
Overall	Total	24 (36%)	17 (28%)	10 (16%)	51
	Not Switched	19 (28%)	9 (15%)	9 (15%)	37 (73%)
	Switched	5 (8%)	8 (13%)	1 (1%)	14 (27%)
	ALT	0	1 (2%)	0	1 (2%)
	USG-PL	0	0	1 (1%)	1 (2%)
	USG-RT	5 (8%)	7 (11%)	0	12 (23%)

We found a significant difference in preparation time in all techniques as P-value < 0.05. No significant difference was found in venous access time, cannulation time, and duration of the procedure (Table 4). On the other hand, we only found a minor complication in eight (4.2%) patients in our study, mainly carotid puncture and hematoma (Table 5).

**Table 4:** Comparison of the mean preparation time, venous assess cannulation time, and duration of the procedure among groups a: ANOVA test ALT, Anatomical landmark technique; USG, Ultrasound guided

Variables	Anatomical landmark (n=67)		Pre-location USG (n=61)		Real-time USG (n=61)		P-value^ a^
	Mean	SD	Mean	SD	Mean	SD	
Preparation time (min)	4.14	1.938	5.58	1.201	5.01	2.029	< 0.001*
Venous access (min)	3.24	2.677	3.68	3.190	3.16	2.816	0.572
Cannulation time (min)	2.61	3.679	2.12	1.296	3.10	3.978	0.252
Duration of procedure (min)	9.81	6.29	11.33	4.25	10.49	4.67	0.259

**Table 5:** Complications occurred among the groups c: chi-square exact test ALT, Anatomical landmark technique; USG, Ultrasound guided

Complications	Total (n=189)	Anatomical landmark (n=67)	Pre-location USG (n=61)	Real-time USG (n=61)	P-value^ c^
Yes	8 (4.23%)	6 (8.96%)	1 (1.64%)	1 (1.64%)	0.058
No	181 (95.8%)	61 (91.0%)	60 (98.4%)	60 (98.4%)	

## Discussion

It has already been documented how ultrasonography may be used for a variety of venous access procedures, including planning access, guiding insertion, and detecting early and late problems [12-14]. When IJV is chosen for central venous cannulation, the American Society of Anesthesiologists' practice guidelines advise using real-time ultrasonography guidance for vascular identification and puncture [7]. The use of ultrasound can lessen difficulties by lowering the frequency of failed entry attempts. An increased risk of pneumothorax, artery puncture, and nerve damage is linked to several needle passes. Additionally, repeated needle passes raise the risk of perivascular hemorrhage or vasospasm, both of which can make cannulating the artery more technically challenging and raise the possibility of catheter-associated thrombus owing to reduced blood flow surrounding the catheter [15,16].

There is no conflict of opinion among anesthesiologists regarding USG placement of IJV catheterization as many studies have been proved about its success and safety [3,4,17]. Our study also showed the same that the success rate is higher in the real-time ultrasound technique, but using ultrasound pre-location technique and anatomical landmark technique has a much lower success rate. As a resource-limited area, most of our anesthesiologists were trained with anatomical landmark techniques, so the anatomical technique in our population is widely used. However, our local studies found the same result in favor of US usage. In our studies, we also got the same result about the anatomical CVC placement as the success rate was only 64.2% in the first attempt. On the other hand, different types of literature were available regarding the number of attempts while cannulating veins successfully for CVC insertion. In this study, we used the first three attempts for any techniques for successful cannulation. Our study showed successful cannulation in 73% compared to 26% that required more than three attempts or switched from the assigned technique to another technique. There is conflicting data regarding the success rate of either applying the short-axis (SAX) or the long-axis (LAX) ultrasound view to the visualization of the vein and its cannulation for CVC insertion; both can be adopted, and success depends on the experience of either technique. When we use SAX to visualize both veins and arteries simultaneously, it will not be easy to perfectly control the tip of the needle. On the other hand, in LAX, the IJV is centered under the probe. The blood vessel and the probe needle are both in the ultrasound beam to easily optimize the needle visualisation. However, in this case, only the vein can be visualised, and any other surrounding anatomical structures cannot be displayed [18].

Regarding complications, only minor complications related to carotid puncture (4.2%) were noted in our study, mostly consistent with other studies [19]. We did not find any significance in venous access time, cannulation time, and duration of the procedure, but only found significance in preparation time only.

In low-income countries such as Pakistan, the availability of ultrasound in most hospitals is limited as the cost is the major limiting factor. The cost can further increase when the real-time technique is used as sterile ultrasound sleeves and jelly are used. This study showed no significant difference in terms of cannulation and complications, and we agreed with Ray et al. that using the pre-location technique will decrease expenditures [20]. However, nothing much we can benefit from this technique if we compare it to real-time US guidance. Differences were found only in preparation time P-value < 0.05, and pre-location took a little higher time in terms of the preparation mean of 5.58 min. Our study did not show any difference in venous access, cannulation, and duration time among all techniques, although our results are different and showed higher time taken in every step for performing CVC. The reasons for the differences in results from previous studies might be the involvement of residents in most of our cases as our institute is a leading teaching institute in Pakistan, although every case is supervised by consultants. This study showed only minor complications, around 4.2%, mainly carotid punctures with little effect only in four patients.

The following are the limitations of this study. Much larger study data are needed as the sample size is small, and as the experience in ultrasound usage was limited, we did not include the whole time consumed for CVC insertion from skin preparation to suturing and dressing. We also did not control the teaching element, which might be the main contributor to time consumption for every step.

## Conclusions

Any technique can be used for IJV cannulation, but the most acceptable is the real-time US technique. However, no difference in the overall procedure time among all three techniques was noted, and no major complications were found.

## References

[1] Boulet N, Muller L, Rickard CM, Lefrant JY, Roger C (2023). How to improve the efficiency and the safety of real-time ultrasound-guided central venous catheterization in 2023: a narrative review. Ann Intensive Care.

[2] Kehagias E, Galanakis N, Tsetis D (2023). Central venous catheters: which, when and how. Br J Radiol.

[3] Brass P, Hellmich M, Kolodziej L, Schick G, Smith AF (2015). Ultrasound guidance versus anatomical landmarks for internal jugular vein catheterization. Cochrane Database Syst Rev.

[4] Kunhahamed MO, Abraham SV, Palatty BU, Krishnan SV, Rajeev PC, Gopinathan V (2019). A comparison of internal jugular vein cannulation by ultrasound-guided and anatomical landmark technique in resource-limited emergency department setting. J Med Ultrasound.

[5] Hermosura B, Vanags L, Dickey MW (1966). Measurement of pressure during intravenous therapy. JAMA.

[6] Lamperti M, Biasucci DG, Disma N (2020). European Society of Anaesthesiology guidelines on peri-operative use of ultrasound-guided for vascular access (PERSEUS vascular access). Eur J Anaesthesiol.

[7] Apfelbaum JL, Rupp SM, Tung A (2020). Practice guidelines for central venous access 2020: an updated report by the American Society of Anesthesiologists task force on central venous access. Anesthesiology.

[8] Ray BR, Mohan VK, Kashyap L, Shende D, Darlong VM, Pandey RK (2013). Internal jugular vein cannulation: a comparison of three techniques. J Anaesthesiol Clin Pharmacol.

[9] Fathi M, Izanloo A, Jahanbakhsh S, Taghavi Gilani M, Majidzadeh A, Sabri Benhangi A, Paravi N (2016). Central venous cannulation of the internal jugular vein using ultrasound-guided and anatomical landmark techniques. Anesth Pain Med.

[10] MF Filho, Lawall P, de Souza KM, Palitot I, Magalhaes I, Vasconcelos H (2013). Comparison between ultrasound-guided and anatomic landmark puncture of the right internal jugular vein. J Cardiovasc Dis Diagn.

[11] Burki LCA, Khan A, Butt B (2012). Comparison between anatomical versus ultrasonographic insertion of central venous line. Pak Armed Forces Med J.

[12] Leung J, Duffy M, Finckh A (2006). Real-time ultrasonographically-guided internal jugular vein catheterization in the emergency department increases success rates and reduces complications: a randomized, prospective study. Ann Emerg Med.

[13] Biasucci DG, La Greca A, Scoppettuolo G, Pittiruti M (2015). What's really new in the field of vascular access? Towards a global use of ultrasound. Intensive Care Med.

[14] Vafek V, Skříšovská T, Kosinová M (2022). Central venous catheter cannulation in pediatric anesthesia and intensive care: a prospective observational trial. Children (Basel).

[15] Sakuraya M, Okano H, Yoshihiro S, Niida S, Kimura K (2022). Insertion site of central venous catheter among hospitalized adult patients: a systematic review and network meta-analysis. Front Med (Lausanne).

[16] Saul T, Doctor M, Kaban NL, Avitabile NC, Siadecki SD, Lewiss RE (2015). The ultrasound-only central venous catheter placement and confirmation procedure. J Ultrasound Med.

[17] Trabelsi B, Hajjej Z, Drira D, Yedes A, Labbene I, Ferjani M, Ben Ali M (2022). Comparison of ultrasound-guided internal jugular vein and supraclavicular subclavian vein catheterization in critically ill patients: a prospective, randomized clinical trial. Ann Intensive Care.

[18] Chaudhary S, Atwal MA (2021). A review of various techniques of central venous catheter insertion. Int J Sci Health Res.

[19] Lal J, Bhardwaj M, Verma M, Bansal T (2020). A prospective, randomised, comparative study to evaluate long axis, short axis and medial oblique axis approach for ultrasound-guided internal jugular vein cannulation. Indian J Anaesth.

[20] Balaban O, Aydin T, Musmul A (2020). Lateral oblique approach for internal jugular vein catheterization: randomized comparison of oblique and short-axis view of ultrasound-guided technique. North Clin Istanb.

